# Future leader to watch – Gregory Redpath

**DOI:** 10.1242/bio.059203

**Published:** 2022-02-02

## Abstract

First Person is a series of interviews with the first authors of a selection of papers published in Biology Open, helping early-career researchers promote themselves alongside their papers. Gregory Redpath is first author on ‘ Serotonin: an overlooked regulator of endocytosis and endosomal sorting?’, published in BiO. Gregory is a postdoc at the Lowy Cancer Research Centre, The University of New South Wales (UNSW) Kensington Campus, Sydney, Australia, investigating the regulation of endocytosis – how cells internalise what they need to function – and in particular how this ties in with serotonin and mental health.


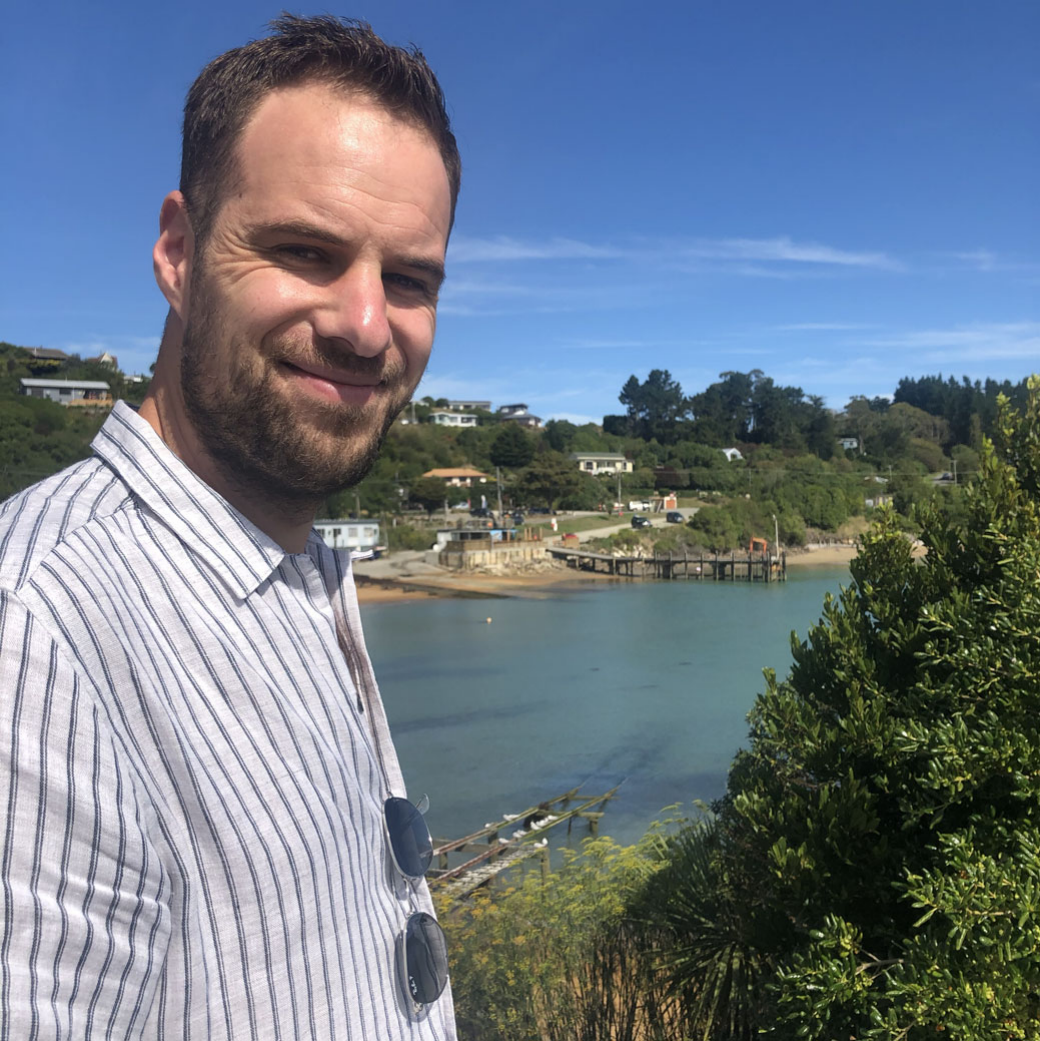


**Gregory Redpath**

**What is your scientific background and the story of how you got to where you are today?**

I began my research career undertaking an Honours project with Professor Sally McCormick at the University of Otago, New Zealand. Following my Honours, I moved to Australia for my PhD, then undertook a postdoctoral position with Dr Jeremie Rossy at the EMBL Australia Node in Single Molecule science at UNSW. This initiated my interest in endocytosis: Dr Rossy introduced me to the visually spectacular and (to me) the very informative world of live cell imaging, photoactivation and optogenetics. After this position, I moved back to New Zealand, where my partner now was, and began studying psychology while working part-time as a postdoc for Prof. McCormick. I quickly realised that while I was very passionate about the psychological sciences, I wanted to research the cell biological basis of mental health conditions, an area that is so often overlooked. I continued working on a lipoprotein project with Prof. McCormick full-time and found some very interesting data that could explain some of the elusive connections between cardiovascular disease and mental health conditions, specifically with regard to lipoprotein metabolism and serotonin. I moved back to Australia in 2020 and began another postdoc with Dr Vaishnavi Ananthanarayanan at UNSW. I have been working with Dr Ananthanarayanan to contribute my knowledge on endocytosis and its intersection with motor proteins, and I am also hoping to pursue what exactly serotonin does with endocytosis.

**What is the most important take-home message of your Review?**

Serotonin, a hormone we all associate with our mood, appears to have some very fundamental, body-wide functions that have long been observed but not well understood. It appears that serotonin may enhance how the cells in our body internalise nutrients to thrive and function by influencing how they take these up from circulation. Moreover, it seems as though there may be multiple different ways that serotonin does this.

“It appears that serotonin may enhance how the cells in our body internalise nutrients to thrive and function by influencing how they take these up from circulation.”

**What has surprised you the most while researching this Review?**

It has long been observed that serotonin can enhance endocytosis with the first studies performed in the 1970s and continuing into the 1980s. Serotonin's effect on mood and mental health, however, has superseded much of the research on its effect on endocytosis. This was surprising to me, given my interest in endocytic processes! Also, the current renaissance of serotonin research highlights its impact throughout the whole body, not just the brain. Serotonin has many functions outside of the brain and I think it is exciting what this could tell us about its impact on our health.

**Figure:**
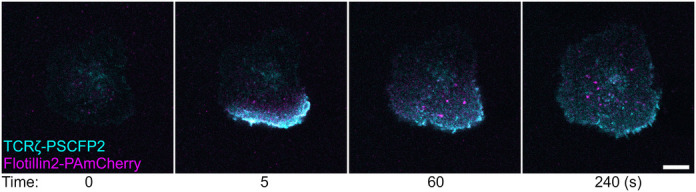
**This image is of some ‘double photoactivation’ experiments I performed in Jurkat T cells.** These experiments never worked particularly consistently as there was no easy way to pick a transfected cell, but when they did work, they looked spectacular! Here, the T cell receptor (TCRζ) is attached to photoswitchable CFP (PSCFP2), and flotillin2 is attached to photoactivatable mCherry. I hit the periphery of the cell with UV light and crossed my fingers that I would see endocytosis of both proteins at once. It worked pretty well in this experiment – you can see an accumulation of TCRζ and flotillin2 endosomes over time, and a lot of TCRζ filtering throughout the plasma membrane.

**What do you feel is the most important question that needs to be answered to move the field forward?**

To me, the most important question is: what are the precise endocytic mechanisms that serotonin governs? It appears that serotonin receptor signalling, conjugation of serotonin onto small GTPases and serotonin insertion into the plasma membrane can all enhance endocytic uptake, but we do not know how these all (if at all) work together to orchestrate endocytosis. Thus far, it appears that serotonin will enhance clathrin-independent endocytic pathways but to know how all these pieces fit together is going to require a precise evaluation of serotonin's impact on each endocytic pathways, which, I believe, can give a powerful glimpse into the way that certain serotonin-modulating medications do and don't work and enhance our fundamental understanding of endocytic processes.

**What changes do you think could improve the professional lives of early-career researchers (ECRs)?**

In Australia, it is definitely down to creating more opportunities for ECRs to obtain small project grants (e.g. 1 year funding). There are generous fellowships, but in lieu of receiving a large fellowship, which can take a couple of years, ECRs need to be able to obtain smaller pots of funding. Getting to establish your proof-of-principle data early on is necessary in not only demonstrating the strength of your ideas but also garner the confidence to keep persisting on what can otherwise be a very challenging start to establishing oneself as an independent researcher.

**What's next for you?**

Like most researchers, I am hoping get some funding! I am also hoping to obtain a fellowship so I can begin establishing my own research programme on serotonin and its role in cellular trafficking and the cellular basis of affective disorders such as depression and anxiety.
